# Solidarität mit Kindern und Menschen mit Behinderung? Eine Wirtschaftlichkeitsanalyse hüftrekonstruierender Eingriffe in der Kinderorthopädie

**DOI:** 10.1007/s00132-023-04381-7

**Published:** 2023-05-17

**Authors:** Katharina Susanne Gather, Sébastien Hagmann, Simone Gantz, Tobias Renkawitz

**Affiliations:** Orthopädische Universitätsklinik Heidelberg, Schlierbacher Landstr. 200a, 69118 Heidelberg, Deutschland

**Keywords:** Zerebralparese, DRG, Hüftluxation, Rekonstruktive chirurgische Verfahren, Behandlungskosten, Cerebral palsy, DRG, Hip dysplasia, Reconstructive surgical procedures, Treatment costs

## Abstract

**Hintergrund und Fragestellung:**

Zerebrale Schädigungen können als infantilen Zerebralparese (ICP) oder durch andere Erkrankungen vorliegen. Durch Störung des Muskeltonus entwickelt sich häufig eine neurogene Hüftdezentrierung. Ein hüftrekonstruierender Eingriff kann die Mobilität und Pflegequalität der Kinder wesentlich verbessern. Derartige operative Versorgungen werden durch Abwertungen im DRG-System finanziell benachteiligt. In der Folge ziehen sich kinderorthopädische Abteilungen aus diesem Versorgungskonzepten zurück, es besteht das Risiko einer Unterversorgung.

**Material und Methode:**

Ziel dieser retrospektiven Studie war eine Wirtschaftlichkeitsanalyse von kinderorthopädischen Eingriffen am Beispiel der neurogenen Hüftdezentrierung. Dazu wurde bei Patienten mit ICP und mit anderen zerebralen Schädigungen die Erlös-Kosten-Situation an einem Haus der Maximalversorgung im Zeitraum 2019–2021 untersucht.

**Ergebnisse:**

Über den gesamten Analysezeitraum zeigt sich eine Verlustsituation. Bei Kindern ohne frühkindliche Hirnschädigung noch deutlicher als in der Gruppe der Patienten mit ICP. Während hüftrekonstruktive Eingriffe bei Kindern mit ICP zunächst 2019 noch kostendeckend zu erbringen waren, sind auch diese Versorgungen seit 2021 defizitär.

**Diskussion:**

Obwohl die Unterscheidung zwischen einer ICP und anderen kindlichen Hirnschäden im kinderorthopädischen Versorgungskonzept keine Rolle spielt, zeigt sich vor allem die nicht-CP-Gruppe im DRG-System deutlich unterfinanziert. Insgesamt offenbart sich die negative wirtschaftliche Bilanz der Kinderorthopädie. Kinder mit Behinderung können in der aktuellen Auslegung des DRG-Systems an einem universitären Zentrum der Maximalversorgung nicht kostendeckend versorgt werden.

## Abstract (en)

**Background:**

Brain damage in childhood can be caused cerebral palsy (CP) or be due to other diseases. Disturbance of muscle tone results in consecutive development of hip subluxation. Hip reconstructive surgery can significantly improve the mobility and quality of care of children. However, the DRG for surgical care of these conditions has been increasingly devalued. In Germany, this has already led to a reduction of pediatric orthopedics departments, accompanied by an important risk of insufficient treatment options for children and people with disabilities.

**Method:**

The aim of this retrospective study was an economic analysis of pediatric orthopedic interventions using the example of neurogenic hip decentration. For this purpose, the revenue–cost situation in patients with CP or other causes of brain damage was evaluated at a maximum care hospital in the period of 2019–2021.

**Results:**

The entire analysis period showed a deficit. The non-CP-group showed the most important deficit. In CP-patients, however, the plus decreased each year and resulted in a deficit in 2021.

**Conclusion:**

While the distinction between cerebral palsy and other forms of brain damage in children is usually not relevant for treatment, it is evident that the non-CP group is massively underfinanced. Overall, the negative economic balance of pediatric orthopedics in the field of neurogenic hip reconstruction is clearly revealed. In the current interpretation of the DRG system, children with disabilities cannot be offered cost-effective care at a maximum-care university center.

**Graphic abstract:**

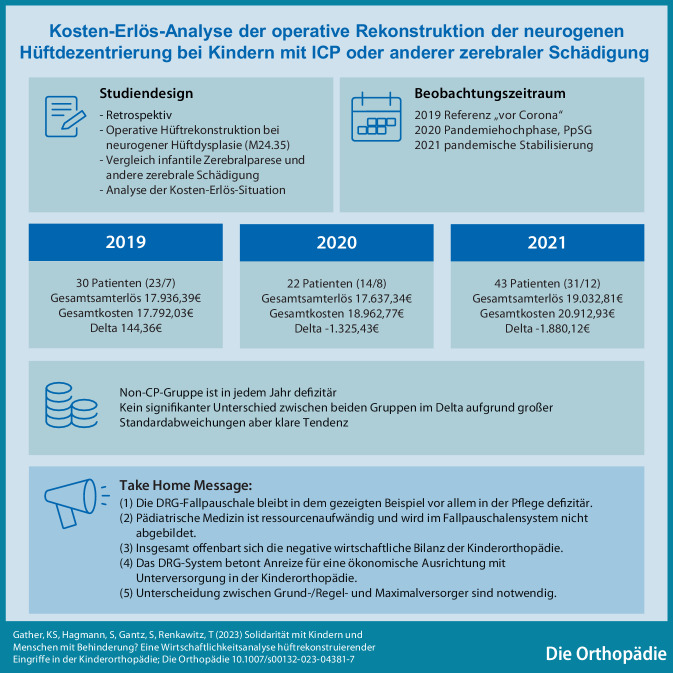

## Hintergrund

Das aktuell gültige, im Jahr 2004 verbindlich eingeführte Abrechnungssystem stationärer Krankenhausleistungen auf Basis diagnosebezogener Fallpauschalen (DRG = Diagnosis Related Groups) betonte von Anfang an Anreize für eine ökonomische Ausrichtung. Der zwangsläufig provozierte Effekt, ökonomischen Aspekten Einfluss auf die Therapieentscheidung zu überlassen, wird insgesamt als Fehlentwicklung bewertet [6]. Gerade die Behandlung von Kindern erfordert dabei einen deutlich höheren personellen, technischen und zeitlichen Aufwand als jene von Erwachsenen. Beispielsweise steigt der Personalkostenaufwand bei der Betreuung von Kindern auf 85 %, während der vergleichbare Aufwand in der Erwachsenenversorgung durchschnittlich 60 % beträgt. Die Besonderheiten der Anforderungen zeigen sich auch in der Abrechnungssystematik. Kommt die Erwachsenenmedizin aktuell mit durchschnittlich 200 Fallpauschalen aus, gibt es in der Pädiatrie derzeit rund 500 Fallpauschalen [12]. Auch der Sachkostenbereich ist bei der Versorgung von Kindern aufwändig. Durch hohe Vorhaltekosten für die gesamte Alterspalette vom Kleinkind bis Jugendlichen und der geringeren Stückzahl, sind die Implantatkosten wesentlich teurer als in der Erwachsenenversorgung [7]. Diese Umstände sind bekannt. Auch die Krankenhausreformkommission der Bundesregierung hat in ihrer ersten Stellungnahme auf eine teilweise dramatische Situation in der Pädiatrie, Kinderchirurgie und Geburtshilfe hingewiesen [8, 11, 13, 15, 16].

**Figure 1:**
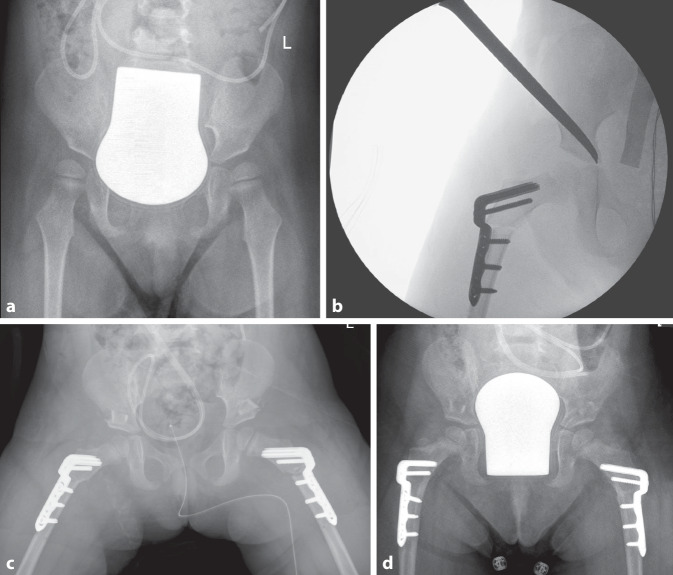

Die Kinderorthopädie beschäftigt sich mit den operativen und nichtoperativen Versorgungen bei kongenitalen Fehlbildungen und Syndromen, der Behebung kindlicher Achs- und Rotationsfehlern, Skelettdysplasien, Wirbelsäulendeformitäten, kindlichem Rheuma und vielen weiteren Entitäten einschließlich der pädiatrischen Neuroorthopädie. Spätestens bei der dritten Vorsorgeuntersuchung (U3) des Säuglings haben junge Eltern zum ersten Mal Kontakt mit der Kinderorthopädie. Eine angeborene Hüftdysplasie kann mithilfe der Hüftsonographie erkannt und oftmals durch nichtoperative Verfahren im Wachstum zur Ausheilung gebracht werden. Bei Kindern mit zerebralen Schädigungen gelingt dies durch die immanente Veränderung des Muskeltonus nicht. In der Folge kommt es zu einer neurogenen Dezentrierung des Hüftgelenks. Betroffene Kinder leiden unter Schmerzen, sind in ihrer Transfermobilität (z. B. für pflegerische Maßnahmen) und Lebensqualität stark eingeschränkt. Mit der chirurgischen Hüftrekonstruktion lässt sich diese Situation grundlegend verbessern (Abb. 1).

Bislang gibt es kaum Wirtschaftlichkeitsanalysen bei kinderorthopädischen Eingriffen. Ziel unserer Arbeit war es daher, die Kosten-Erlös-Situation an einem universitären Haus der Maximalversorgung anhand dieses häufigen Eingriffs, der operativen Hüftrekonstruktion bei Kindern mit Zerebralparese und anderer zerebraler Schädigung zu vergleichen.

Der Grund für die Auswahl des letztgenannten Unterscheidungsmerkmals soll im Folgenden kurz erklärt werden. Für das DRG-System ist zur Diagnosestellung der ICP das Eintreten eines „frühkindlichen“ Hirnschadens entscheidend. Derzeit wird das Neugeborenen- und Säuglingsalter als bemessungsrelevanter Zeitpunkt definiert. Wenn also ein Kind zu früh auf die Welt kommt, und infolge eines Sauerstoffmangels intrauterin einen Hirnschaden mit einer spastischen Tetraparese erleidet, so wird bei diesem Kind im Alter von 6 Jahren eine ICP diagnostiziert und verschlüsselt. Hätte ein Kind andererseits mit 4 Jahren einen schweren Unfall und erleidet dabei durch Sauerstoffunterversorgung einen Hirnschaden, der in einer spastischen Tetraparese resultiert, so wird dieses Kind der Definition nach im Alter von 6 Jahren nicht in die Diagnosegruppe der ICP verschlüsselt. Das kinderorthopädische Therapiekonzept ist aufgrund der gleichen Symptomatik für beide Kinder identisch. Sie haben häufig exakt die gleichen orthopädischen Symptome, und die gleiche Hüftsubluxation. Eine Operation und stationäre Versorgung zieht bei beiden Kindern den gleichen Aufwand nach sich. Allerdings triggern sie unterschiedliche DRGs. Die Auswirkungen auf die Vergütung soll im Folgenden betrachtet werden.

## Methodik

Es wurden retrospektiv und monozentrisch die Fälle aus den Jahren 2019–2021 verglichen, bei denen die Hauptdiagnose einer neurogenen Hüft(sub-)luxation (M24.35) sowie die Nebendiagnose einer ICP (G80.x, CP-Gruppe) oder andere zerebrale Schädigungen (non-CP-Gruppe) vorlagen, und die Prozedur einer Derotationsvarisationsverkürzungsosteotomie (DVO) des Femurs (5–781.6f) in Kombination mit einer Azetabuloplastik nach Dega (5–829.00) und teilweise auch einer offene Einstellung der Hüfte (Kapselraffung 5–807.0, Eröffnen der Gelenkkapsel 5–800.0G) durchgeführt wurde. Bezüglich der Gruppe der non-CP-Patienten sind die Diagnosen vielfältig und im Folgenden aufgeführt: Pyruvatdehydrogenasemangel (E74.4), schwere Hirnstammstörung mit Dauerbeatmung (Q04.3), kongenitale Glykosierungsstörung (E77.0), Rett-Syndrom (F84.2), Prader-Willi-Syndrom (Q87.1), Aicardi-Syndrom (Q04.0), Lesch-Nyhan-Syndrom (E79.1), Cri-du-chat-Syndrom (Q93.4), Wolf-Hirschhorn-Syndrom (Q93.3), Tubulinopathie (Q04.3), Pelizaeus-Merzbacher-Erkrankung (E75.2), Karboanhydrasemangel (E88.8). Ausgeschlossen wurden Diagnosen ohne direkte primäre zerebrale Schädigung wie Myelomeningozele (Q05.2/Q05.9; MMC, Spina bifida), Arthrogryposis (Q74.3), „Lower-limb“-Amyoplasie (Q65.1) oder Trisomie 21 (Q90.9). Grundsätzlich können diese Diagnosen auch das Gehirn betreffen, beeinflussen von ihrer Genese jedoch nicht ausschließlich durch eine Hirnschädigung den Muskeltonus.

Es wurden zur Einsicht die entsprechenden im Controlling (Verband der Universitätsklinika Deutschlands e. V. [VUD] Kalkulationsdaten, QlikView x64, QlikTech GmbH, Düsseldorf, Deutschland) und Medizincontrolling (SAP DRG-Arbeitsplatz, SAP Deutschland SE & Co. KG, Walldorf, Deutschland, und 3M^TM^ 360 Encompass^TM^, 3M Deutschland GmbH, Neuss, Deutschland) genutzten Abrechnungsprogramme der Universitätsklinik Heidelberg verwendet. Hierbei sind die Ist-Kosten die realen Fallkosten des Hauses und die Standardkosten die vom InEK (Institut für das Entgeltsystem im Krankenhaus) kalkulierten Kosten für diese Fallart. Aufgeschlüsselt wurden Kosten für Ärzte, Pflege, medizinisch-technischen Dienst, Arzneimittel, Implantate, medizinischen Bedarf, Fremdkosten, medizinische Infrastruktur und allgemeine Infrastruktur.

Zur Erhebung des Gesamterlöses wurden der DRG-Erlös und die Zu- oder Abschläge sowie die Zusatzentgelte erhoben, ab 2020 auch das herausgelöste Pflegeentgelt. Die Abrechnungsgrundlage sind die von den Operateuren postoperativ verschlüsselten Diagnosen und Prozeduren, der Operationsbericht sowie der stationäre Entlassbrief.

Erhoben wurden die zugrundeliegenden Diagnosen, Alter zum Operationszeitpunkt, intraoperative additive Maßnahmen, Schnitt-Naht-Zeit, Operationsseite, Verweildauer auf Intensiv- und Normalstation, Schweregradgruppierung, Pflegeentgelt, Gesamterlös, DRG-Erlös, Zusatzentgelte, Zu- und Abschläge, Gesamt-Ist- und Standardkosten aufgeschlüsselt nach Kostenarten- und Kostenstellengruppe.

Alle Jahrgänge werden separat erhoben und analysiert. Die Auswertung erfolgte mit SPSS (IBM SPSS Statistics, Version 28.0.0, IBM Deutschland GmbH, Ehningen, Deutschland). Zur Bildung von Mittelwerten und Standardabweichungen wurde eine deskriptive Statistik verwendet. Da beide Untergruppen (CP- und non-CP-Gruppe) voneinander unabhängig waren, wurde für die Subgruppenvergleiche für metrische Daten der T‑Test für unabhängige Stichproben genutzt. Der Levene-Test gab hier Aufschluss über Varianzhomogenität oder Varianzheterogenität. Für den Vergleich aller 3 Jahre wurde die ANOVA-Analyse als Erweiterung des T‑Testes (Levene-Test) als zweifaktorielle Varianzanalyse verwendet.

## Ergebnisse

Es wurde der Verlauf über 3 Jahre ausgewertet. 2019 wurden insgesamt 30, 2020 insgesamt 22 und 2021 43 Patienten mit operativer Versorgung an der Orthopädischen Universitätsklinik Heidelberg, entsprechend den erfüllten Kriterien eingeschlossen und ausgewertet.

Die in den einzelnen Jahren abgerechneten DRG sind in Tab. 1 zusammengefasst und beschrieben. Von 2019 zu 2020 kam es zu einer deutlichen Reduktion der Bewertungsrelation [3]. Die Basisfallwerte entwickelten sich in Baden-Württemberg mit 3539,12 € zum 01.03.2019, 3672,40 € zum 01.02.2020 und 3763,00 € zum 01.03.2021 [14].

**Table 1:** 

DRG	Bezeichnung	Bewertungsrelation		
		2019	2020	2021
A09C	Beatmung > 499 h oder > 249 h mit intensivmedizinischer Komplexbehandlung > 2352/1932/2208 P., mit komplexer OR-Prozedur oder Polytrauma oder intensiver Komplexbehandlung > 1764/1656/2208 P. oder mit komplizierender Konstellation oder Alter < 16 Jahre	18,372	12,298	12,306
B17B	Eingriffe an peripheren Nerven, Hirnnerven und anderen Teilen des Nervensystems oder Eingriffe bei zerebralen Lähmung, Muskeldystr. od. Neurop., mit best. kompl. Eingr., Alter < 16 J. oder mit mäßig kompl. Eingr., Alter < 19 J. oder mit äuß. schw. oder schw. CC	2456	1816	1735
I08B	Andere Eingriffe an Hüftgelenk und Femur mit sehr komplexem Eingriff bei komplexer Diagnose oder äußerst schweren CC	4642	3626	3987
I08C	Andere Eingriffe an Hüftgelenk und Femur mit Einbringen von Abstandshaltern oder and. komplexen Eingriffen bei kompl. Diagnose od. äußerst schweren CC	3598	3103	3059
I08D	Andere Eingriffe an Hüftgelenk und Femur mit komplexer Diagnose oder mit komplexer Prozedur oder mit äußerst schweren CC	2852	2309	2507
I08E	Andere Eingriffe an Hüftgelenk und Femur ohne komplexe Diagnose oder Prozedur, ohne äußerst schwere CC, mit bestimmten Eingriffen an Becken und Femur oder mit bestimmten komplizierenden Diagnosen	2637	2169	2216
I08F	Andere Eingriffe an Hüftgelenk und Femur ohne Eingr. in Komb. Hüftgel. und obere Extremität oder Wirbelsäule, ohne best. kompliz. Faktoren, mit best. and. Eingr. an Hüftgel. und Femur oder best. kompl. Eingr. Femur und Becken ohne best. Diagn., > 1 BT	1954	1555	1535
I26Z	Intensivmedizinische Komplexbehandlung > 588/552/552 Aufwandspunkte bei Krankheiten und Störungen an Muskel-Skelett-System und Bindegewebe oder hochaufwendiges Implantat bei hochkomplexer Gewebe‑/Hauttransplantation	10,767	7797	7978
I37Z	Resezierender Eingriff am Becken bei bösartiger Neubildung des Beckens oder Mehretageneingriffe an der unteren Extremität	4353	3718	3643

*BewR* Bewertungsrelation, *BT* Belegungstag, *CC* Complication or Comorbidity, *DRG* Diagnosis Related Groups

**Figure 2:**
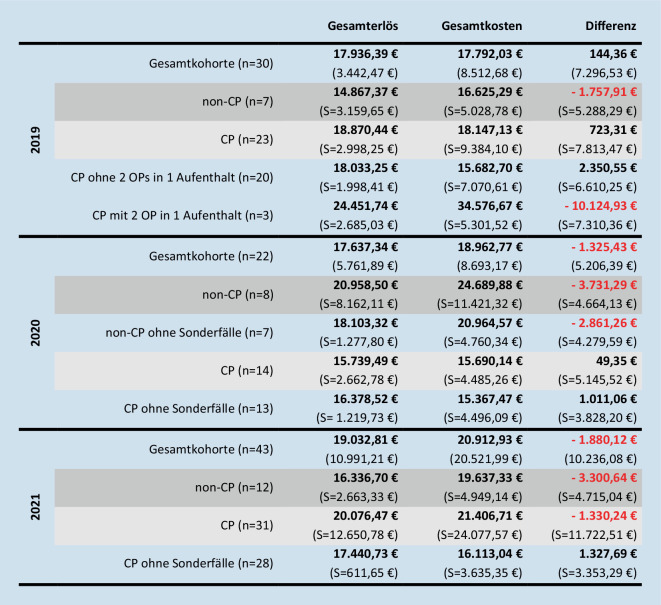

Zur Übersicht sind die Kohorteneigenschaften in Tab. 2 und die Kosten-Erlös-Situation der Jahre 2019–2021 in der Abb. 2 zusammengefasst. Über alle Jahre ist ersichtlich, dass sie sich hinsichtlich ihrer Eigenschaften nicht unterscheiden und daher vergleichbar sind.

**Table 2:** 

	2019				2020				2021			
	Gesamt	CP	Non-CP	*p*-Wert	Gesamt	CP	Non-CP	*p*-Wert	Gesamt	CP	Non-CP	*p*-Wert
Anzahl	30 [27]	23 [20]	7	–	22 [20]	14 [13]	8 [7]	–	43 [40]	31 [28]	12	–
Alter (a)	7,0 (3,4) [7,3 (3,4)]	7,4 (3,1) [8,0 (2,9)]	5,6 (4,1)	0,210 [0,112]	6,4 (3,3) [6,3 (3,2)]	6,9 (3,5) [7,1 (3,6)]	5,6 (3,1) [4,7 (1,8)]	0,402 [0,119]	6,77 (3,6) [6,7 (3,4)]	7,1 (3,6) [7,0 (3,3)]	5,9 (3,6)	0,339 [0,356]
Geschlecht (m:w)	17:13 [16:11]	13:10 [12:8]	4:3	0,977 [0,943]	13:9 [11:9]	7:7 [6:7]	6:2 [5:2]	0,251 [0,517]	21:2 [18:22]	17:14 [14:14]	4:8 [4:8]	0,206 [0,332]
GMFCS-Level												
I	–	1 [0]	–	–	–	1	–	–	–	1	–	–
II	–	4	–	–	–	1	–	–	–	3	–	–
III	–	3	–	–	–	3	–	–	–	6	–	–
IV	–	8 [6]	–	–	–	2	–	–	–	5	–	–
V	–	7	–	–	–	7 [6]	–	–	–	15 [12]	–	–
Operationszeit	149,0 (36,1) [150,6 (36,3)]	155,2 (37,6) [158,3 (37,6)]	128,7 (21,9)	0,089 [0,063]	146,8 (53,2) [154,6 (49,39)]	144,3 (54,8) [150,2 (52,3)]	151,1 (53,5) [162,7 (45,7)]	0,780 [0,600]	150,7 (47,6) [147,3 (46,9)]	151,4 (54,4) [146,6 (54,3)]	148,8 (23,8)	0,875 [0,894]
Operationsprozedur				0,400 [0,305]				0,262 [0,274]				0,228 [0,426]
DVO + Dega	17 [16]	14 [13]	3		20 [18]	12 [11]	8 [7]		30	20	10	
DVO + Dega + offen	13 [11]	9 [7]	4		2	2	–		13 [10]	11 [8]	2	
Verweildauer (d)	18,3 (9,4) [16,0 (6,5)]	19,0 (10,6) [16,0 (7,5)]	16,0 (2,9)	0,236 [1,000]	13,3 (3,9) [12,7 (2,8)]	11,8 (2,5) [11,7 (2,6)]	15,9 (4,7) [14,4 (2,4)]	**0,014** **[0,034]**	15,4 (11,6) [13,3 (3,8)]	15,7 (13,5) [12,7 (3,6)]	14,7 (4,0)	0,802 [0,132]
ITS-Aufenthalt (n)	14 [12]	10 [8]	4	0,096 [0,150]	10	4 [3]	6 [5]	0,152 [0,148]	39 [37]	27 [25]	12	0,246 [0,352]
Verweildauer ITS (d)	1,3 (2,3) [1,4 (2,4)]	0,8 (1,4) [0,9 (1,5)]	3,0 (3,7)	0,170 [0,176]	1,6 (2,5) [1,5 (2,5)]	0,6 (1,4) [0,3 (0,6)]	3,4 (3,1) [3,6 (3,3)]	0,440 [**0,040**]	1,4 (1,2)	1,2 (0,9) [1,1 (0,84)]	1,9 (1,6)	0,150 [0,141]
Schweregrad CC				**0,020** **[0,025]**				0,361 [0,224]				0,636 [0,591]
0	2 [2]	–	2		2 [1]	1 [–]	1		–	–	–	
1	–	–	–		–	–	–		–	–	–	
2	–	–	–		1	–	1		8	7	1	
3	15 [14]	14 [13]	1		11 [10]	6	5 [4]		16 [15]	10 [9]	6	
4	8 [7]	5 [4]	3		7	6	1		15 [14]	11 [10]	4	
5	5 [4]	4 [3]	1		1	1	–		4 [3]	3 [2]	1	

*M* männlich, *w* weiblich, *DVO* Derotationsvarisationsosteotomie, *Dega* Acetabuloplastik nach Dega, *GMFCS* Gross Motor Functions Classification System, *ITS* Intensivstation, *offen* offene Hüftrekonstruktion, *d* Tage, *a* Jahre

### Ergebnisse für das Jahr 2019

Signifikante Unterschiede lagen bei den Schweregraden (*p* = 0,020) und abgerechneten DRGs (*p* = 0,012) vor. Der Gesamterlös unterschied sich signifikant in beiden Gruppen (*p* = 0,015) und war in der Non-CP-Gruppe deutlich geringer bei sich nicht unterscheidenden Gesamtkosten in beiden Gruppen. Dies führte im Mittel zu Verlusten von −1757,91 € in der Non-CP-Gruppe und bei der CP-Gruppe zu einem Gewinn von 723,31 €. Dieser Unterschied war allerdings nicht signifikant.

Im Jahr 2019 waren drei Fälle auffällig. Hier wurde innerhalb eines Aufenthaltes zunächst die eine Seite und 7–10 Tage später die andere Seite operiert. Ohne diese besonderen Fälle zeigt sich ebenfalls ein signifikanter Unterschied im Gesamterlös (*p* = 0,005). Nicht unterschieden sich die Gesamt-Ist-Kosten und die Differenz zwischen Erlös und Kosten (*p* = 0,151). Die CP-Gruppe zeigte einen deutlich höheren Gewinn mit 2350,56 €, während die Non-CP-Gruppe unverändert im negativen Bereich mit −1757,91 € blieb.

### Ergebnisse für das Jahr 2020

Im coronageprägten Jahr 2020 zeigte sich an allen deutschen Universitätskliniken, der Trend eines Rückgangs der Operationszahlen. Kinderorthopädischen Operationen wurden an der Orthopädischen Universitätsklinik Heidelberg Priorität eingeräumt, die Anzahl der auswertbaren Fälle reduzierten sich deshalb mit 22 Fällen geringer als in anderen kinderorthopädischen Zentren der Bundesrepublik.

Der Gesamterlös war in der Non-CP-Gruppe deutlich höher (20.958,59 €) als in der CP-Gruppe (15.739,49 €, *p* = 0,037). Jedoch lagen die Gesamtkosten aufgrund längerer Intensivstationsaufenthalte in der Non-CP-Gruppe (24.689,88 € versus 15.690,14 €, *p* = 0,064) höher. Dadurch unterschied sich die Kosten-Erlös-Differenz nicht (*p* = 0,102), obwohl die Non-CP-Gruppe im Mittel mit −3731,29 € Verluste und die CP-Gruppe mit 49,35 € Gewinn verbucht.

Alle Standardkostengruppen des InEK unterschieden sich signifikant in beiden Gruppen. Die Gesamtkosten zeigten jedoch keinen signifikanten Unterschied und überwogen weiterhin in der Non-CP-Gruppe (20.964,57 € versus 15.367,46 €, *p* = 0,018). Der Gesamterlös blieb in der Non-CP-Gruppe mit 20.958,59 € versus 15.739,49 € (*p* = 0,037) höher als in der CP-Gruppe. Weiterhin war die Kosten-Erlös-Differenz in der Non-CP-Gruppe (−2861,26 €) negativ, und in der CP-Gruppe deutlich positiv (1011,06 €, *p* = 0,053).

Auch 2020 waren zwei Fälle in der Erhebung auffällig. Sie waren zum einen durch eine hohe effektive Bewertungsrelation von 7,797 und zum anderen durch eine sehr geringe effektive Bewertungsrelation von 1,55 gekennzeichnet.

### Ergebnisse für das Jahr 2021

Die Kostenverlustrechnung beider Gruppen zeigte in diesem Jahr keinen signifikanten Unterschied (*p* = 0,091). Bei höheren Gesamtkosten der CP-Gruppe zeigte diese einen geringeren Verlust mit −1330,24 € als in der Non-CP-Gruppe (−3300,64 €). Bei den Implantatkosten bestand ein deutlicher Unterschied (*p* = 0,041).

Auch im Jahr 2021 sind 3 Fälle der CP-Gruppe auffällig. Hierbei handelt es sich um einen Patienten, der extern in die Kinderneurologie entlassen wurde (Abschlag von −6502,50 €), und im zweiten Fall als Langlieger 73 Tage stationär war (effektive Bewertungsrelation mit 7,702 und Zuschlag von 22.453,77 €). Der dritte Fall war ein Patient mit komplikationsbehaftetem Verlauf durch postoperative Aspiration und Entwicklung eines Lungenversagens.

Der DRG-Erlös unterschied sich im Jahr 2021 in beiden Gruppen deutlich (*p* = 0,047). Der Gesamterlös (inklusive Pflegeentgelt und Zu‑/Abschläge) unterschied sich jedoch nicht. Die Kosten der Non-CP-Gruppe lag im Mittel über denen der CP-Gruppe. Hier zeigte sich in den Arztkosten (*p* = 0,004), Pflegekosten (*p* = 0,003) und Kosten für medizinischen Bedarf (*p* = 0,027) ein eindeutiger Unterschied (Gesamtkosten CP-Gruppe 16.113,04 €, Non-CP-Gruppe 19.637,33 €, *p* = 0,016). Bei gleichem Gesamterlös zeigte sich hierdurch ein Verlust von −3300,64 € in der Non-CP-Gruppe und ein Gewinn von 1327,69 € in der CP-Gruppe (*p* = 0,001).

### Vergleich der Kosten-Erlös-Entwicklung innerhalb des Analysezeitraumes

Die Abb. 2 fasst die Ergebnisse getrennt nach Gruppen mit und ohne genannte Sonderfälle zusammen. Die Analyse des DRG-Erlöses der CP-Gruppe ergab über die Jahre einen deutlichen Unterschied (*p* = 0,032). Zunächst fiel der DRG-Erlös im Mittel um 3647 €, stieg im Folgejahr wieder um 2797,21 € an. Für den Gesamterlös und die Gesamtkosten zeigte sich eine ähnliche Dynamik, allerdings ohne signifikante Unterschiede. Die Gesamtkosten reduzierten sich von 2019 zu 2020 um durchschnittlich 2456,59 €, stiegen anschließend 2021 jedoch relevant um 5716,57 € an. Dadurch fiel die Kosten-Erlös-Differenz 2020 um 673,96 € und 2021 um 1379,59 €. Keine signifikanten Unterschiede ergab dieselbe Kostenanalyse innerhalb der Non-CP-Gruppe. Hier lagen die Gesamtkosten konstant über dem Gesamterlös, sodass die Kosten-Erlös-Differenz in allen Jahren ausgeprägt negativ blieb. Zunächst betrug der Verlust im Mittel 1973,38 € und verringerte sich im Folgejahr gering um 430,65 €.

Rechnet man für alle Jahre die Sonderfälle heraus, so werden in der CP-Gruppe die Unterschiede aufgrund der reduzierten Standardabweichung im DRG-Erlös eindeutiger mit *p* < 0,001 und im Gesamterlös mit *p* = 0,004. Ohne Sonderfälle mit ihren besonderen Kosten fällt die Differenz zunächst um 1339,49 € und steigt im Jahr 2021 um 316,63 €. Weiterhin bestanden keine Unterschiede im Verlauf der Jahre für die Non-CP-Gruppe.

Vergleicht man weitere Parameter, wie die Verweildauern, Operationszeit und Intensivliegezeit über die Jahre 2019–2021, zeigt sich kein Unterschied. Lediglich in der CP-Gruppe zeigte sich eine deutliche Reduktion der Verweildauer von 2019 zu 2020 mit *p* = 0,004. Analysiert man erneut ohne Sonderfälle, zeigt sich ein signifikanter Unterschied in der Verweildauer der CP-Gruppe (*p* = 0,041).

## Diskussion

Die in dieser Arbeit analysierten Zeiträume sind – geprägt durch die Corona-Pandemie ab März 2020 – besonders zu interpretieren. 2019 kann in gewissem Maße als Referenz „vor Corona“ gelten, wenngleich es von 2019 zu 2020 zu einer deutlichen Abwertung der Bewertungsrelationen (Tab. 1) der berechneten DRGs, auch durch Herauslösen des Pflegeentgeltes gemäß des 2019 in Kraft tretenden Pflegepersonal-Stärkungsgesetzes (PpSG), kam [1, 2, 9]. Einen wesentlichen Unterschied im Pflegeentgelt gab es zwischen den Gruppen nicht.

Bei dem beckenrekonstruktiven Eingriff wird perioperativ ein Periduralkatheter verwandt, um dem Anspruch an ein bestmögliches Schmerzmanagement gerecht zu werden. Analog den Empfehlungen internationaler Fachgesellschaften wird in diesem Zusammenhang eine 24-stündige kontinuierliche Überwachung des Blutdrucks, EKG und der Sauerstoffsättigung auf der Intensivstation gewährleistet [5].

Die Behandlung von Kindern und Jugendlichen erfolgt zunehmend nicht mehr in spezialisierten stationären Einrichtungen, sondern wird von vielen Häusern aus Kostengründen auf Normalstationen „miterledigt“. Es werden Stationen verkleinert oder gar ganz geschlossen. Die damit verbundenen Kapazitätsreduktion aus wirtschaftlichen Gründen führt im Bereich der Kinderorthopädie zu einer Überlastung in den wenigen verbliebenen, kinderorthopädischen Zentren der Bundesrepublik [7]. Eine aktuell durchgeführte Umfrage der „Kommission Kinderorthopädie“ der Deutschen Gesellschaft für Orthopädie und Orthopädische Chirurgie (DGOOC) zeigt, dass mittlerweile auch an Universitätskliniken die Unterfinanzierung das kinderorthopädische Versorgungsangebot beschränkt [17]. In der vorgelegten Analyse zeigt sich beispielhaft, dass die Versorgung der neurogenen Hüftdezentrierung bei behinderten Kindern ohne die Klassifikation als „frühkindlicher“ Hirnschaden im DRG-System für die erbringende Klinik durchgängig mit einem relevanten ökonomischen Defizit verbunden ist. Die Argumentationskette, dass Mehrerlöse bei anderen Verfahren Verluste bei minderfinanzierten Verfahren im DRG-System kompensieren sollen, verfängt sich in der Realität der Betrachtung: Auch bei einem lebensqualitätsverbessernden Eingriff bei Kindern mit ICP resultiert für die erbringende Abteilung seit 2021 ein Verlust. Die Entwertung kinderorthopädischer Eingriffe, beispielhaft gezeigt an der Hüfteinstellung bei neurologisch erkrankten Kindern, hat in den letzten Jahren zu einem reflexartigen Rückzug von Krankenhausträgern aus diesem Versorgungsbereich geführt. Die Gefahr einer Unterversorgung der kleinsten und schwächsten Patienten mit orthopädischen Problemen ist keine Theorie mehr. Wartezeiten für ambulante Vorstellungstermine in den verbliebenen kinderorthopädischen Zentren von teilweise über einem Jahr sind mittlerweile die Folge [10].

Auffällig ist die große Differenz hinsichtlich der InEK-kalkulierten und tatsächlichen Kosten für Pflege und Ärzte. Über den gesamten Analysezeitraum liegen die Kosten für die Pflege relevant über den in der Abrechnungssystematik hinterlegten Kosten. Zum Teil lässt sich dies möglicherweise auf die Tatsache zurückzuführen, dass die Analyse in einem orthopädisch-universitären Zentrum der Maximalversorgung durchgeführt wurde. Universitätskliniken haben aufgrund des häufig sehr kranken und aufwendigen Patienten sowie der gesetzlich vorgesehenen Aufgaben in Forschung, Lehre, Aus- und Weiterbildung höhere Kostenaufwendungen für die Behandlung. Standardisierungen und Prozessoptimierungen können die Effektivität und den Kostenaufwand in der Krankenversorgung grundsätzlich reduzieren. Die über 400 verschiedenen kinderorthopädischen Erkrankungen sind dem Grunde nach nahezu alle seltene Erkrankungen und damit kaum standardisierbar. Kinder mit seltenen Erkrankungen profitieren dabei von einer hohen Behandlungsqualität spezialisierter Ärzte [4, 7].

Die bekannte Regel, dass Komplikationen grundsätzlich wahrscheinlicher sind, je kränker ein Patient ist, gilt in der Erwachsenen- und Kindermedizin gleichermaßen. Die kinderorthopädische Behandlung erfordert deshalb häufig hohe Interdisziplinarität und längere Verweildauern [4]. Dies erfordert Investitionen in Ausstattung, Medizinbedarf, Intensivkapazität und Infrastruktur. Die DRG-Fallpauschale bleibt in dem gezeigten Beispiel an allen Stellen defizitär. Das Ergebnis dieser wirtschaftlichen Analyse ist dabei keine Ausnahme, sondern ein Beispiel für die ökonomische Realität der kinderorthopädischen Versorgung in Deutschland. Die Autoren möchten mit der vorgelegten Auswertung denen eine Stimme geben, die sich selbst nicht äußern können: Den kleinsten und schwächsten Patienten unserer Gesellschaft.

## Fazit für die Praxis

- Das DRG-System (DRG: Diagnosis Related Groups) betont Anreize für eine ökonomische Ausrichtung.
- Die DRG-Fallpauschale bleibt in dem gezeigten Beispiel defizitär.
- Pädiatrische Medizin ist ressourcenaufwändig und wird im Fallpauschalensystem nicht abgebildet.
- Insgesamt offenbart sich die negative wirtschaftliche Bilanz der Kinderorthopädie.
- Ohne Gegensteuerung droht eine Unterversorgung im kinderorthopädischen Bereich.

## Abkürzungen

BewR: Bewertungsrelation
CC: Complication or Comorbidity
DRG: Diagnosis Related Groups
DVO: Derotationsvarisationsverkürzungsosteotomie
ICP: infantile Zerebralparese
InEK: Institut für das Entgeltsystem im Krankenhaus
PpSG: Pflegepersonal-Stärkungsgesetz
VUD: Verband der Universitätsklinika Deutschlands e. V.
